# Multimodal deep learning radiomics model for predicting postoperative progression in solid stage I non-small cell lung cancer

**DOI:** 10.1186/s40644-024-00783-8

**Published:** 2024-10-17

**Authors:** Qionglian Kuang, Bao Feng, Kuncai Xu, Yehang Chen, Xiaojuan Chen, Xiaobei Duan, Xiaoyan Lei, Xiangmeng Chen, Kunwei Li, Wansheng Long

**Affiliations:** 1 Department of Radiology, Hainan General Hospital, 19#, Xiuhua Road, Xiuying District, Haikou, Hainan Province 570311 PR China; 2 Laboratory of Artificial Intelligence of Biomedicine, Guilin University of Aerospace Technology, Guilin City, Guangxi Province 541004 China; 3 Department of Radiology, Jiangmen Central Hospital, 23#, North Road, Pengjiang Zone, Jiangmen, Guangdong Province 529030 PR China; 4 Department of Nuclear Medicine, Jiangmen Central Hospital, Jiangmen, Guangdong Province 529030 PR China; 5 Department of Radiology, The Fifth Affiliated Hospital of Sun Yat-sen University, Zhuhai, Guangdong Province 519000 PR China

**Keywords:** Non-small cell lung cancer, Deep learning, Postoperative progress, Computed tomography, Radiomics

## Abstract

**Purpose:**

To explore the application value of a multimodal deep learning radiomics (MDLR) model in predicting the risk status of postoperative progression in solid stage I non-small cell lung cancer (NSCLC).

**Materials and Methods:**

A total of 459 patients with histologically confirmed solid stage I NSCLC who underwent surgical resection in our institution from January 2014 to September 2019 were reviewed retrospectively. At another medical center, 104 patients were reviewed as an external validation cohort according to the same criteria. A univariate analysis was conducted on the clinicopathological characteristics and subjective CT findings of the progression and non-progression groups. The clinicopathological characteristics and subjective CT findings that exhibited significant differences were used as input variables for the extreme learning machine (ELM) classifier to construct the clinical model. We used the transfer learning strategy to train the ResNet18 model, used the model to extract deep learning features from all CT images, and then used the ELM classifier to classify the deep learning features to obtain the deep learning signature (DLS). A MDLR model incorporating clinicopathological characteristics, subjective CT findings and DLS was constructed. The diagnostic efficiencies of the clinical model, DLS model and MDLR model were evaluated by the area under the curve (AUC).

**Results:**

Univariate analysis indicated that size (*p* = 0.004), neuron-specific enolase (NSE) (*p* = 0.03), carbohydrate antigen 19 − 9 (CA199) (*p* = 0.003), and pathological stage (*p* = 0.027) were significantly associated with the progression of solid stage I NSCLC after surgery. Therefore, these clinical characteristics were incorporated into the clinical model to predict the risk of progression in postoperative solid-stage NSCLC patients. A total of 294 deep learning features with nonzero coefficients were selected. The DLS in the progressive group was (0.721 ± 0.371), which was higher than that in the nonprogressive group (0.113 ± 0.350) (*p* < 0.001). The combination of size、NSE、CA199、pathological stage and DLS demonstrated the superior performance in differentiating postoperative progression status. The AUC of the MDLR model was 0.885 (95% confidence interval [CI]: 0.842–0.927), higher than that of the clinical model (0.675 (95% CI: 0.599–0.752)) and DLS model (0.882 (95% CI: 0.835–0.929)). The DeLong test and decision in curve analysis revealed that the MDLR model was the most predictive and clinically useful model.

**Conclusion:**

MDLR model is effective in predicting the risk of postoperative progression of solid stage I NSCLC, and it is helpful for the treatment and follow-up of solid stage I NSCLC patients.

**Graphical abstract:**

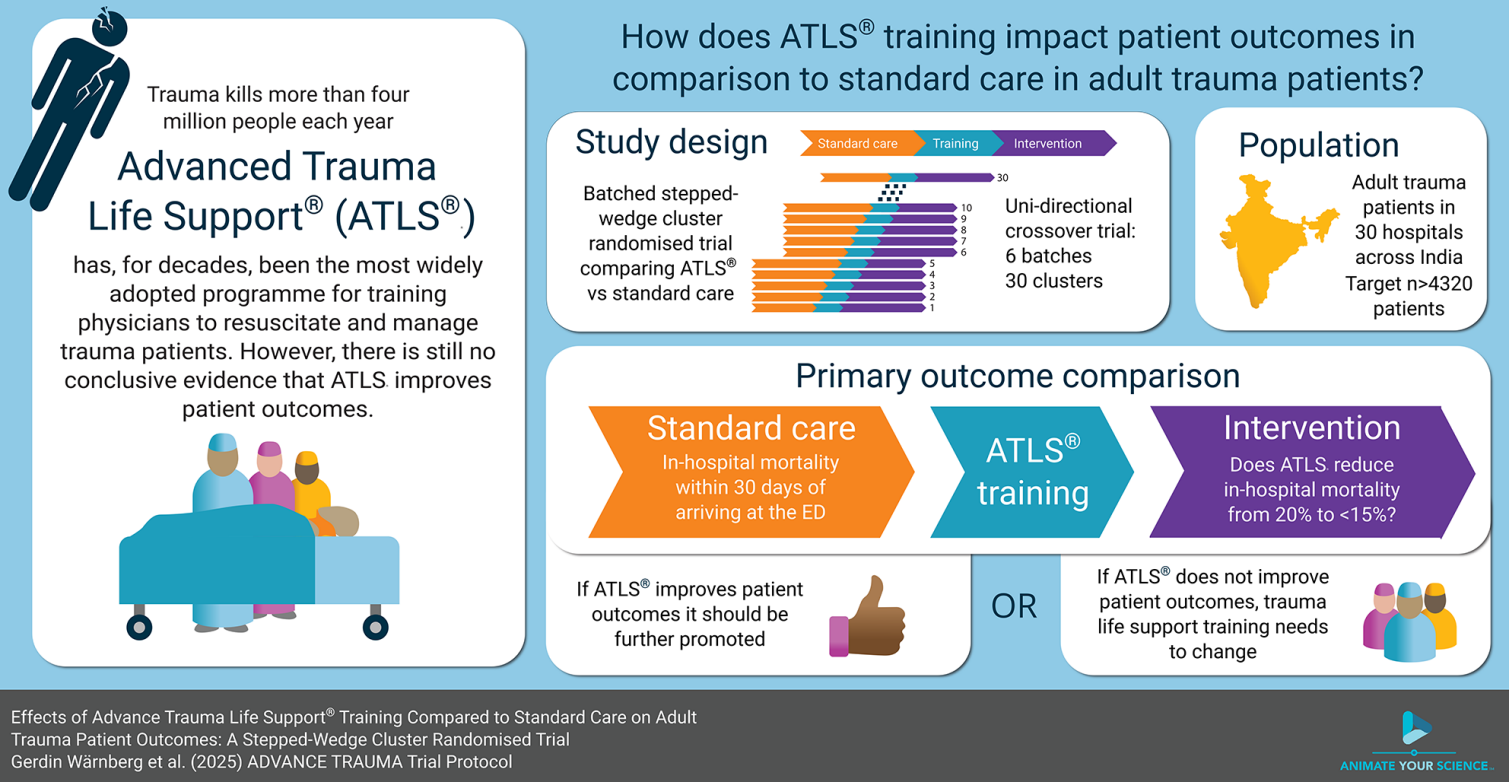

**Supplementary Information:**

The online version contains supplementary material available at 10.1186/s40644-024-00783-8.

## Background

Lung cancer is the leading cause of cancer death [1]. Non-small cell lung cancer (NSCLC) is the most common pathological type of lung cancer, accounting for approximately 85% of the total incidence of lung cancer [2]. According to the National Comprehensive Cancer Network (NCCN) guidelines, anatomical lobectomy and lymph node dissection are the main surgical treatments for patients with NSCLC [3]. The postoperative progression of lung cancer is closely related to the pathological type and TNM stage. Stage I-III lung adenocarcinoma has a recurrence rate of 18-34.4% [4]. TNM stage is the most important prognostic factor for predicting the recurrence rate and survival time of lung cancer [5]. However, TNM stage also has limitations, and considerable heterogeneity exists within the same stage group. Some patients survive without recurrence for a long time, while others progress rapidly (recurrence or metastasis). Therefore, a more scientific and accurate method is needed to predict the postoperative recurrence and metastasis of lung cancer.

Although surgical resection is the most appropriate choice of treatment for early stage NSCLC and patients with stage I disease have the most favourable prognosis, the recurrence rate of stage I NSCLC has been reported to be 18.5–20.1% [6, 7]. The prognosis may be different with the attenuation of lung cancer nodules. The results showed that there was a significant difference in 5-year overall survival (OS) between the subsolid group and solid group (stage IA1: 97.8% vs. 86.6%, *p* = 0.026; IA2: 89.3% vs. 75.2%, *p* = 0.007; IA3: 88.5% vs. 62.3%, *p* = 0.003) [8]. The results indicated that in the clinical study of stage I NSCLC, the attenuation classification of lung cancer nodules needs to be strictly distinguished. The most common model for predicting postoperative progression (recurrence or metastasis) of stage I lung cancer is the clinical model, which includes histological differentiation and serum tumour biomarkers [9, 10]. Meanwhile, radiological methods such as chest CT findings play an important role in predicting the prognosis of early resected NSCLC. Recurrence was significantly correlated with tumour size, mass type, lobulated sign and peritumoral interstitial thickening [11].

With the emergence of artificial intelligence, traditional machine learning has gradually been used to predict the prognosis of patients. Several studies have shown that the prediction efficiency of a machine learning model combined with a clinical model is higher than that of a single machine learning model or single clinical model in predicting the survival of NSCLC patients [12, 13]. With the rapid development of computers, deep learning (DL) technology has shown great advantages in a variety of complex tasks. Deep learning methods demonstrate a remarkable capability for learning distinctive features, effectively extracting profound features from image data relevant to specific tasks. This ability contributes to achieving higher accuracy in disease diagnosis [14]. Compared with traditional machine learning, DL has more optimized algorithms, richer information extraction and higher specificity. In recent years, DL has been increasingly used in a variety of clinical studies, such as the detection of breast cancer on breast X-rays [15, 16], segmentation of brain tumours using magnetic resonance imaging (MRI) [17, 18], and classification of interstitial lung diseases using high-resolution chest CT. DL has also been applied to the study of survival neural network models and showed potential benefits in prognostic assessment and treatment recommendations of lung cancer-specific survival rates [19, 20].

At present, there are relatively few studies using DL methods to predict the postoperative progression of stage I NSCLC based on chest CT images [21]. In this study, we intend to use DL technology to extract valuable DL features from preoperative CT images of patients with solid stage I NSCLC, integrate clinical and CT findings, and build and verify a joint prediction model. Ancillary objectives are to explore the clinical application value of the multimodal DL radiomics (MDLR) model in predicting the postoperative progression of solid stage I NSCLC and to improve stratified management and precision treatment for patients with solid stage I NSCLC.

## Methods

### Study population

This study was approved by the institutional review board, and informed consent was waived given that this was a retrospective study. Clinical and imaging data of patients with solid NSCLC diagnosed by histopathology after radical surgical resection in our hospital from January 2014 to September 2019 were retrospectively reviewed. The inclusion criteria included the following: (1) patients with solid NSCLC who were surgically resected and confirmed by histopathology; (2) chest CT examination was performed within 1 month before surgery; (3) CT images could be downloaded from the picture archiving and communication system (PACS); (4) regular and complete follow-up records after surgery (at least 3 years); (5) the TNM pathological stage was stage I; and (6) the slice thickness of CT images was less than or equal to 1.5 mm. The exclusion criteria were as follows: (1) preoperative radiotherapy and chemotherapy; (2) patients with other malignant tumours; and (3) patients who were lost to follow-up.

**Fig. 1:**
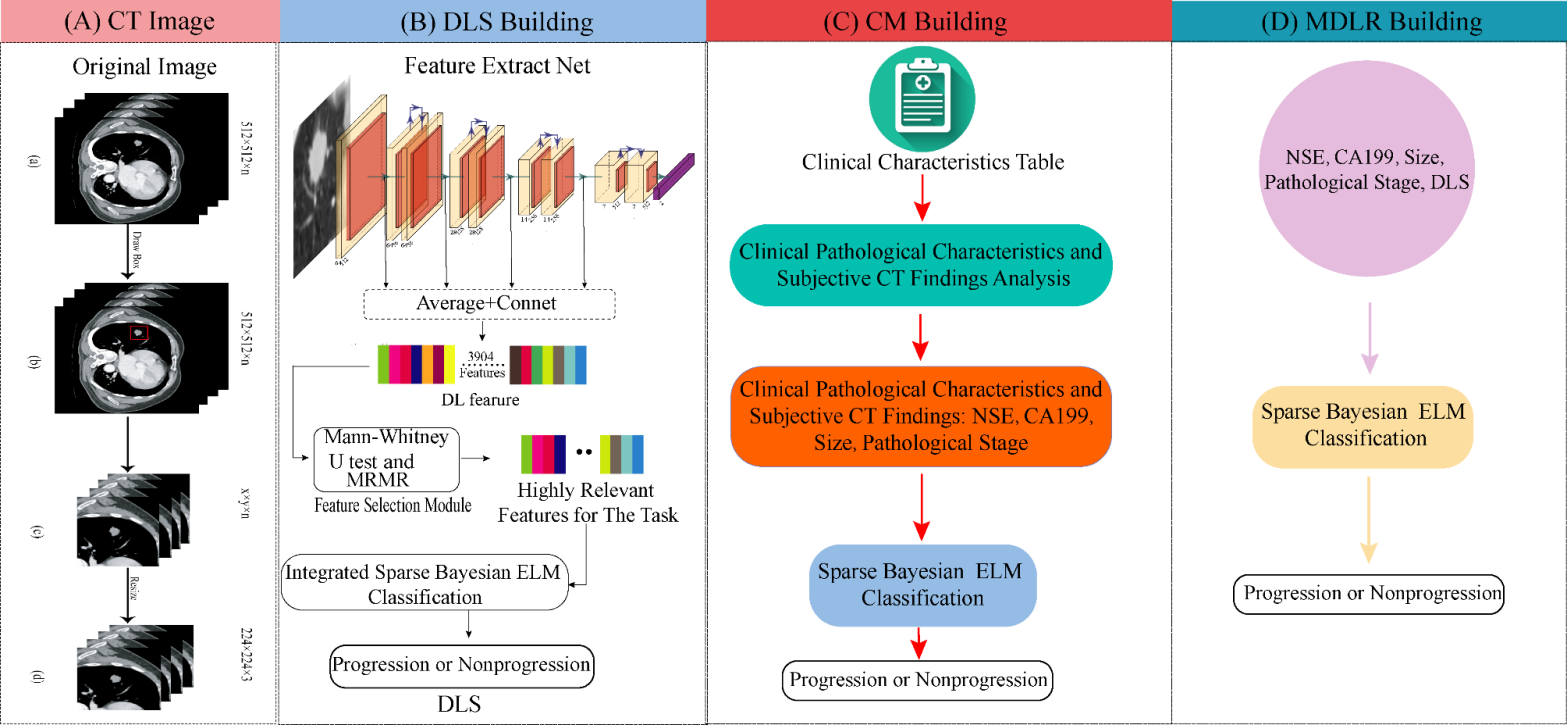
Overall flow chart of the study. (**A**) The ROI extraction process, (**B**) DLS building process, (**C**) CM building process, (**D**) MDLR building process. ROI: region of interest; DLS: deep learning signature; CM: clinical model; MDLR: multimodal deep learning radiomics

A total of 459 patients with solid stage I NSCLC met the study requirements (male, 239; female, 220; mean age, 60.24 ± 10.20 years; range, 21–84 years). There were 72 cases in the progressive group and 387 cases in the nonprogressive group. The patients were randomly allocated to the training cohort and the internal validation cohort at a ratio of 7:3. A total of 321 patients were included in the training cohort, and 138 patients were included in the internal validation cohort. A total of 104 cases (male, 54; female, 50; mean age, 57.13 ± 9.91 years; range, 36–76 years) were collected from another hospital according to the same criteria as an external validation cohort, including 15 cases in the progressive group and 89 cases in the nonprogressive group. Figure 1

### Overall flow chart of the study

#### Chest CT scan protocol and CT finding evaluation

Chest CT scans were conducted by one of the following scanners: Definition Force (Siemens, German), Siemens 16 (Siemens, German), Toshiba Aqilion (Toshiba, Japan), and GE Discovery 64 (GE, America). Spiral CT volume scanning technology was adopted, and the scanning parameters are detailed in Supplementary S1. Subjective CT findings evaluation was performed by two radiologists (with 10 and 15 years of experience in chest imaging diagnosis) independently evaluating the CT findings of lung cancer lesions as detailed in Supplementary S2.

### Clinical information record and follow-up plan

Stage I included stages IA and IB according to the International Association for the Study of Lung Cancer (IASLC) 8th edition TNM stage [22]. The clinical and pathological information of patients was recorded, including smoking history, pathological types, operation method, pathological stage and serum tumour markers. The follow-up plan was as follows: (1) chest CT was reviewed every 6–12 months for the first 2 years after surgery and once a year thereafter; (2) if there were clinical symptoms, the corresponding site was examined; and (3) the end point of the study was the progression of the disease. Patients with no progression were followed up for 3 years or more. The definition of postoperative progress in 3 years of lung cancer was according to prior studies [23] and is detailed in Supplementary S3.

### Data preprocessing

The data used in the pulmonary nodule CT image dataset were approved by the hospital ethics committee, and the privacy protection of patients met the requirements of the regulations. The dataset adopts the original data in digital imaging and communications in medicine (DICOM) format, image matrix 512 × 512, without any modification, editing or lossy compression. The images of each case were kept continuous and complete, without missing layers or split layers.

**Fig. 2:**
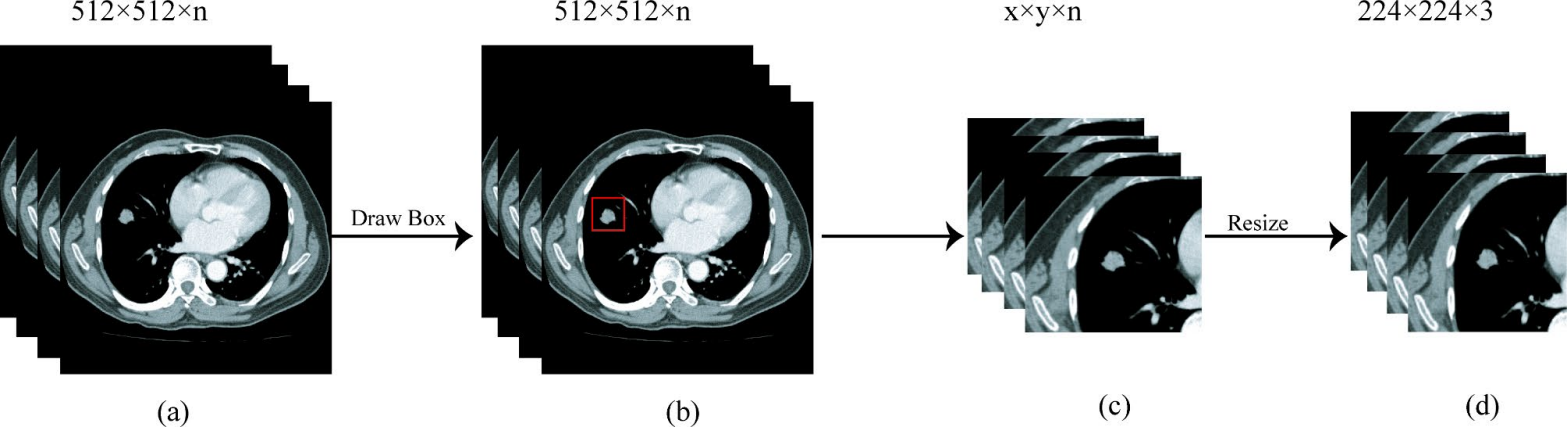
Chest CT image preprocessing. First, we selected all consecutive transverse slices of venous-phase CT images of solid pulmonary nodule lesions (**a**); second, a rectangular bounding box containing the whole region of the lesion was drawn in CT images by reader 1 using our in-house method developed based on MATLAB 2016 (**b**); third, this rectangular bounding box was applied to other layers of the lesion to crop all CT images (**c**); finally, these images were resized to 224* × *224 (**d**); among them, *n* represents the number of slices of a lesion

Venous phase images with a continuous cross-sectional plane were used. The DL model input CT image regions of interest (ROIs) data were constructed as follows: the ROIs were delineated by radiologists with ten years of experience in chest diagnosis. The process involved identifying the location and contour of the lesion, then constructing a rectangular box based on the lesion’s contour that encompasses the entire boundary of the lesion. Consequently, the ROI includes both the entire lesion information and the surrounding tumor information. Therefore, the selection of ROI is less influenced by the subjective experience of the clinicians and does not require the radiologists to precisely delineate the tumor boundaries. For detailed steps, please refer to Fig. 2.

### Building the DLS

To prevent overfitting, this study employed a transfer learning strategy during the training of the DL model. Pretrained ResNet 18 [24] network parameters on the ImageNet dataset were used as the initial model. Subsequently, a fine-tuning strategy was applied, where the gradients of the convolutional layers in the first two layers of ResNet 18 were frozen, and the model was adjusted using the preprocessed images of solid NSCLC, categorized into progression and non-progression groups from this study until the training epochs reached the specified parameters, at which point the model training was stopped. The DL experimental equipment was shown in Supplementary S4.

**Fig. 3:**
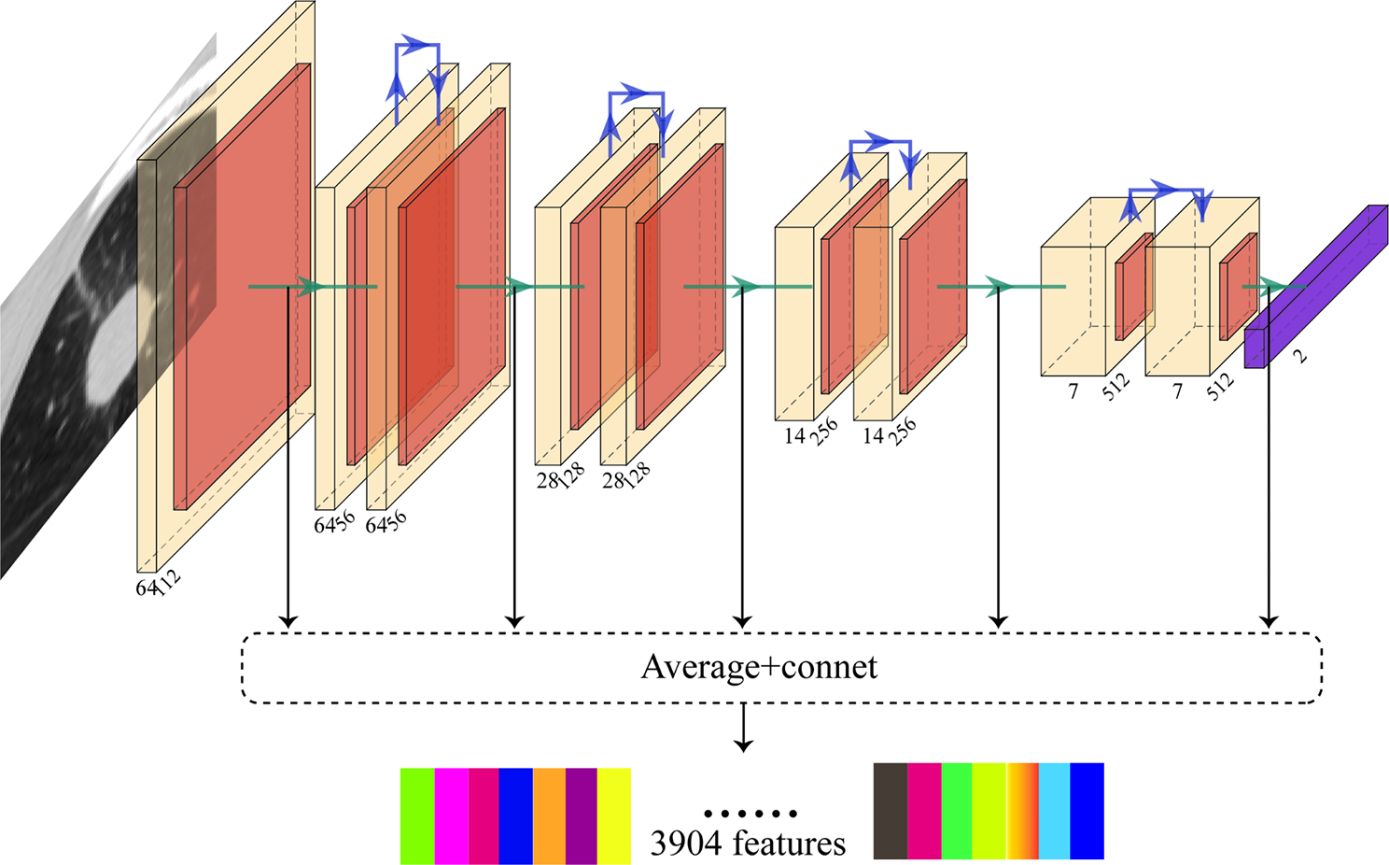
Deep learning feature extraction. First, the preprocessed pictures were input into the trained deep learning model; second, the convolution kernel was used as the feature extractor to average the eigenvalues obtained after the picture passed through each convolution kernel; finally, 3, 904 deep learning features were extracted from each patient by stitching the eigenvalues extracted from each convolution kernel

Due to the redundancy of features in DL, it can lead to overfitting of the model’s classification performance. Therefore, in this study, a ResNet18 network was used as a feature extraction network. The convolutional kernels in the network’s convolutional layers were used as feature extractors, with each kernel corresponding to a DL feature. Subsequently, feature extraction was performed on all images for each patient, and the DL features extracted from all images were averaged to obtain a set of DL features for a single patient. The ResNet18 network contained a total of 3, 904 convolutional kernels, resulting in 3, 904 DL features being extracted for each patient. The feature extraction process of DL is shown in Fig. 3. The detailed parameters of DL training is shown in Supplementary S5.

To further reduce computational complexity and identify highly relevant features for the task, this study employed feature selection techniques such as the U test and the maximum relevanceon the initial set of 3, 904 deep features. Subsequently, the selected features were used to construct an extreme learning machine (ELM) based on integrated strategy classifier (Supplementary S6). This process ultimately allowed for the classification of early lung cancer progression and nonprogression, thereby creating a DL signature.

### Building MDLR model

To perform a comprehensive analysis of the postoperative progression risk status in NSCLC, the following three steps were performed in the training cohort to build a MDLR model that combined DLS, clinical pathological characteristics, and subjective CT findings. First, we used Cohen’s kappa test to analyse the subjective CT findings between the two radiologists, with values of poor (0.00–0.20), fair (0.21–0.40), moderate (0.41–0.60), substantial (0.61–0.80), and near-perfect agreement (0.81–1.00). Second, the Wilcoxon rank-sum test, Fisher’s exact test, or Pearson’s Chi-square test was used to compare clinical variables between groups (gender and age), subjective CT finding and DLS. Finally, the factors with significant differences were selected as inputs for the ELM classifier to construct the MDLR model, enabling a robust analysis of the postoperative progression risk.

### Statistical analysis

R3.0.1 (http://www.rproject.org) and Python 3.6 were used for all statistical analyses. The ROC analyses and decision curve analysis (DCA) were performed using “pROC,” and “dca.r,” respectively. A t test was used to compare age and longest diameter between the progressive and nonprogressive groups. Pearson’s Chi-square test was used to compare gender, emphysema, margin, lobulated sign, speculated sign, vacuole sign, air bronchogram sign, surgical type, pathological stage, smoking history, NSE, Cyfra21-1, CEA and CA199. Fisher’s exact test was used to compare the location and pathological type. Clinical and pathological factors and subjective CT findings were used to construct the clinical model through univariate analysis and ELM.

To assess the performance of each diagnostic model, the sensitivity, specificity, negative predictive value (NPV), positive predictive value (PPV), and accuracy were determined using receiver operating characteristic (ROC) curve analysis. The DeLong test was used to compare the area under the curve (AUC) of the models. The MDLR of the validation and training datasets was evaluated by calibration. By quantifying the net benefit of patients at various threshold probabilities in the cohort, DCA was used to assess the clinical usefulness of the predictive models. *P* < 0.05 on both sides was regarded as statistically significant.

## Results

### Clinicopathologic analysis of solid stage I NSCLC

In the training dataset, 27 males and 23 females were in the progressive group, and 136 males and 135 females were in the nonprogressive group, without a significant difference (*p* = 0.620). The mean ages of the progressive and nonprogressive groups were ((59.9 ± 11.43) years and (60.51 ± 10.40) years, respectively (*p* = 0.725). Univariate analysis showed statistically significant differences in the distribution of pathological stages, NSE and CA199 between the progressive group and the nonprogressive group (*p* = 0.027; *p* = 0.03; *p* = 0.003). There was no significant difference in the distribution of gender, age, pathological type, surgical method, smoking, Cyfra21-1 or CEA between the progressive group and the nonprogressive group (all *p* > 0.05) (Table 1).

**Table 1:** Comparative analysis of clinicopathologic characteristics and subjective CT findings of the patients with stage I solid NSCLC

	Training cohort (n = 321)		*P* value	Internal validation cohort (n = 138)		*P* value	External validation cohort (n = 104)		*P* value
	Progres-sive (n = 50)	Nonpro-gressive (n = 271)		Progres-sive (n = 22)	Nonpro-gressive (n = 116)		Progres-sive (n = 15)	Nonpro-gressive (n = 89)	
**Sex**									
Male	27	136	0.620	16	60	0.069	8	46	0.906
Female	23	135		6	56		7	43	
**Age** (years, mean ± SD)	59.90 ± 11.43	60.51 ± 10.40	0.725	62.14 ± 9.75	59.37 ± 9.26	0.229	54.73 ± 9.20	57.53 ± 9.93	0.317
**Smoking**									
Present	16	63	0.187	9	26	0.068	6	31	0.699
Absent	34	208		13	90		9	58	
**NSE**									
Elevated	7	15	0.03	10	15	< 0.001	0	1	1.000
Normal	43	256		12	101		15	88	
**Cyfra 21 − 1**									
Elevated	13	67	0.848	2	20	0.527	1	2	0.376
Normal	37	204		20	96		14	87	
**CEA**									
Elevated	12	54	0.513	6	5	< 0.001	3	16	1.000
Normal	38	217		16	111		12	73	
**CA199**									
Elevated	7	10	0.003	2	23	0.366	0	2	1.000
Normal	43	261		20	93		15	87	
**Location**	0.423		0.883		0.484				
LUL	17	74		6	27		5	17	
LLL	3	43		4	21		3	13	
RUL	16	82		7	36		5	30	
RML	5	27		2	7		0	11	
RLL	9	45		3	25		2	18	
**Emphysema**									
Present	16	80	0.725	4	21	0.993	2	9	1.000
Absent	34	191		18	95		13	80	
**Size (cm)**	2.64 ± 1.06	2.16 ± 0.96	0.004	2.51 ± 0.99	1.99 ± 0.91	0.032	2.18 ± 0.86	1.95 ± 0.93	0.376
**Margin**									
Irregular	43	234	0.948	22	103	0.128	14	76	0.671
Regular	7	37		0	13		1	13	
**Lobulated sign**									
Present	42	225	0.866	21	100	0.308	13	64	0.375
Absent	8	46		1	16		2	25	
**Speculated sign**									
Present	30	132	0.142	14	72	0.889	7	32	0.428
Absent	20	139		8	44		8	57	
**Vacuole sign**									
Present	8	29	0.281	4	9	0.125	3	8	0.407
Absent	42	242		18	107		12	81	
**Air bronchogram sign**									
Present	16	65	0.231	3	30	0.282	2	19	0.713
Absent	34	206		19	86		13	70	
**Type**									
Adenocarcin-oma	45	234	0.367	22	109	0.655	11	79	0.165
Squamous carcinoma	4	35		0	6		4	8	
other	1	2		0	1		0	2	
**Surgical method**									
Lobectomy	43	240	0.607	9	18	0.006	13	74	1.000
Sub-lobecto-my	7	31		13	98		2	15	
**Pathological stage**									
IA	29	199	0.027	15	89	0.394	9	70	0.216
IB	21	72		7	27		6	19	
**DLS**	0.721 ± 371	0.113 ± 0.350	< 0.001	0.343 ± 0.130	0.117 ± 0.143	< 0.001	0.474 ± 0.142	0.225 ± 0.194	< 0.001

*Notes* NSCLC, non-small cell lung cancer; SD, standard deviation; LUL, left upper lobe; LLL, left lower lobe; RUL, right upper lobe; RML, right middle lobe; RLL, right lower lobe; NSE, neuron-specific enolase; Cyfra 21 − 1, cytokeratin 19 fragment; CEA, carcinoma embryonic antigen; CA199, carbohydrate antigen 19 − 9; DLS, deep learning signature

The comparative analysis of CT findings between the progressive group and the nonprogressive group is shown in Table 1. The tumour size in the progressive group was (2.64 ± 1.06) cm, which was larger than that in the nonprogressive group (2.16 ± 0.96) cm (*p* = 0.004). In the univariate analysis, the distributions of tumour size in the progressive and nonprogressive groups was statistically significant (*p* = 0.004). There were no significant differences in the distribution of location, emphysema, margin, lobulated sign, air bronchogram signs, speculated sign or vacuole sign between the progressive and nonprogressive groups (all *p* > 0.05).

Regarding the training cohort, the AUC of the clinical model based on these four factors was 0.675 (95% CI (confidence interval): 0.599–0.752), and the sensitivity, specificity, accuracy, positive predictive value (PPV) and negative predictive value (NPV) were 82.0%, 46.9%, 52.3%, 22.2% and 93.4%, respectively (Table 2).

**Table 2:** Diagnostic efficiencies of the three predictive models in the training, internal and external cohorts

Datasets	Model	AUC (95% CI)	Sensitivity	Specificity	Accuracy	PPV	NPV
**Training cohort** (*n* = 321)	**Clinical Model**	0.675 (0.599–0.752)	0.820 (41/50)	0.469 (127/271)	0.523 (168/321)	0.222 (41/185)	0.934 (127/136)
	**DLS**	0.882 (0.835–0.929)	0.840 (42/50)	0.757 (205/271)	0.770 (247/321)	0.389 (42/108)	0.962 (205/213)
	**MDLR**	0.885 (0.842–0.927)	0.880 (44/50)	0.720 (195/271)	0.745 (239/321)	0.367 (44/120)	0.970 (195/201)
**Internal validation cohort** (*n* = 138)	**Clinical Model**	0.632 (0.515–0.750)	0.636 (14/22)	0.612 (71/116)	0.616 (85/138)	0.237 (14/59)	0.899 (71/79)
	**DLS**	0.873 (0.803–0.943)	0.455 (10/22)	0.957 (111/116)	0.877 (121/138)	0.667 (10/15)	0.902 (111/123)
	**MDLR**	0.901 (0.839–0.962)	0.727 (16/22)	0.897 (104/116)	0.870 (120/138)	0.571 (16/28)	0.946 (104/110)
**External validation cohort** (*n* = 104)	**Clinical Model**	0.628 (0.482–0.773)	0.533 (8/15)	0.573 (51/89)	0.567 (59/104)	0.174 (8/46)	0.879 (51/58)
	**DLS**	0.852 (0.765–0.939)	0.667 (10/15)	0.798 (71/89)	0.779 (81/104)	0.357 (10/28)	0.934 (71/76)
	**MDLR**	0.861 (0.778–0.945)	0.800 (12/15)	0.719 (64/89)	0.731 (76/104)	0.324 (12/37)	0.955 (64/67)

*Note* AUC, area under the curve; PPV, positive predictive value; NPV, negative predictive value; CI, confidence interval; DLS, deep learning signature; MDLR, multimodal deep learning radiomics

### DLS construction

In the study, a total of 294 DL features were selected from the initial set of 3, 904 deep features using the U test feature selection methods. These 294 DL features were found to be highly relevant to the task while minimizing redundancy. The set of 294 selected DL features was used to construct a classification model using the ELM algorithm. This classification model was then used to generate DLS for the progression and nonprogression groups. These DLS are indicative of the model’s predictions for whether a patient’s condition is progressing or not.

**Fig. 4:**
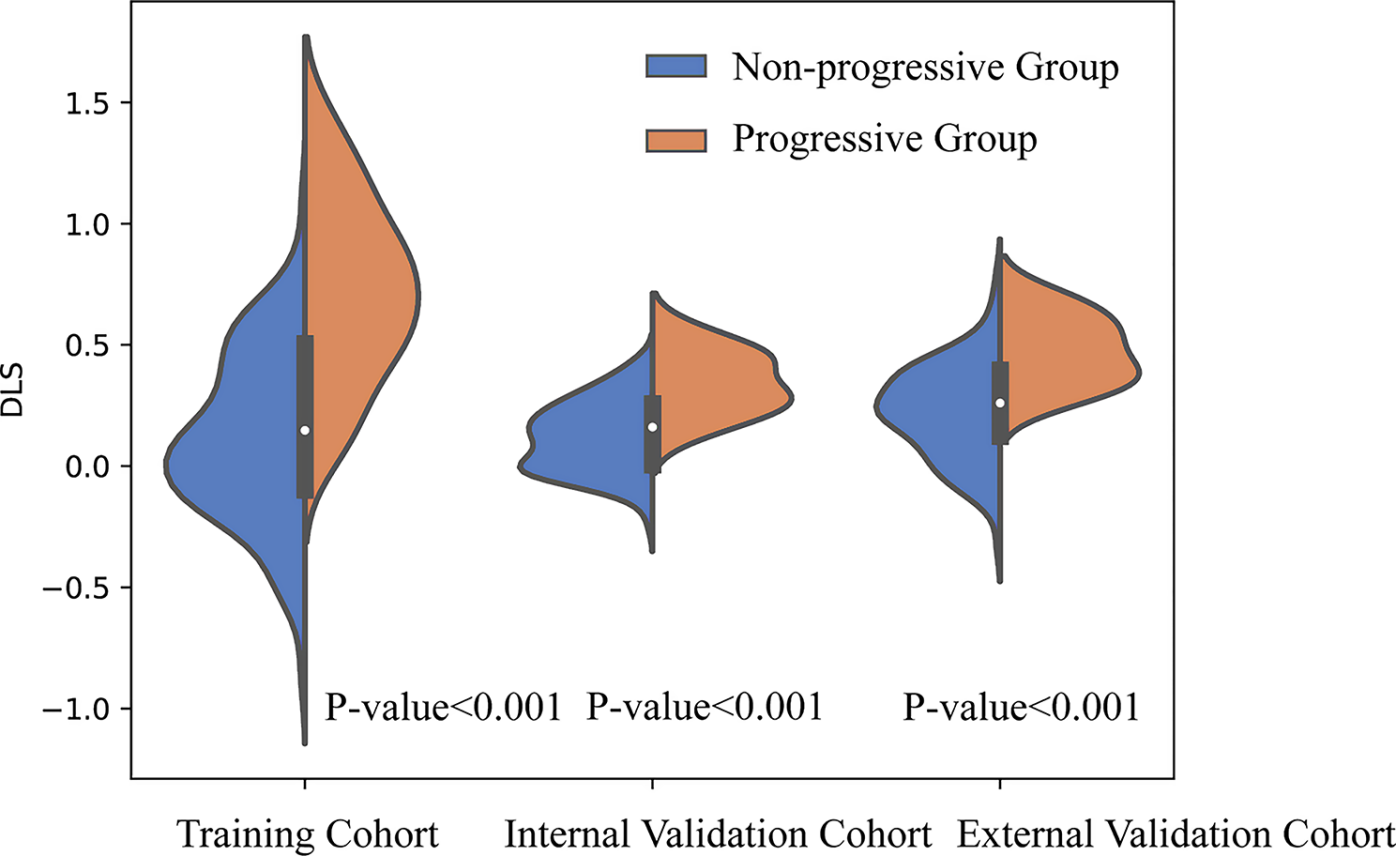
The distribution of DLS in the training, internal and external validation cohorts. DLS, deep learning signature

Regarding the training cohort, the DLS in the progressive group was (0.721 ± 0.371), which was higher than that in the nonprogressive group (0.113 ± 0.350) (*p* < 0.001). Regarding the internal and external validation cohorts (both *p* < 0.001), the DLS in the progressive group were (0.343 ± 0.130) and (0.474 ± 0.142), which were higher than those in the nonprogressive group (0.117 ± 0.143) and (0.225 ± 0.194) respectively (Fig. 4). Regarding the training cohort, the AUC of the deep learning model (DLS model) was 0.882 (95% CI: 0.835–0.929). The sensitivity, specificity, accuracy, PPV and NPV of the DLS model were 84.0%, 75.7%, 77.0%, 38.9% and 96.2%, respectively (Table 2).

To assess the effectiveness of the transfer learning strategy in addressing the imbalance between progressive and non-progressive groups, we constructed a DLS model without using the transfer learning strategy. This model achieved AUC values of 0.843 (95% CI: 0.785–0.899), 0.728 (95% CI: 0.625–0.831), and 0.730 (95% CI: 0.584–0.877) on the training, internal validation, and external validation cohorts, respectively (Table 3), indicating a degree of overfitting. Additionally, the performance of this model across all cohorts was inferior to that of the DLS constructed using the transfer learning strategy. These results demonstrated that employing a transfer learning strategy enhances the model’s applicability and reliability in clinical practice, particularly in the context of imbalanced data.

**Table 3:** DLS and non-transfer learning DLS in the training, internal and external cohorts

Datasets	Method	AUC (95% CI)	Sensitivity	Specificity	Accuracy	PPV	NPV
**Training cohort** (*n* = 321)	DLS	0.882 (0.835–0.929)	0.840 (42/50)	0.757 (205/271)	0.770 (247/321)	0.389 (42/108)	0.962 (205/213)
	Non-transfer learning DLS	0.843 (0.785–0.899)	0.720 (36/50)	0.808 (219/271)	0.794 (255/321)	0.409 (36/88)	0.940 (219/233)
**Internal validation cohort** (*n* = 138)	DLS	0.873 (0.803–0.943)	0.455 (10/22)	0.957 (111/116)	0.877 (121/138)	0.667 (10/15)	0.902 (111/123)
	Non-transfer learning DLS	0.728 (0.625–0.831)	0.455 (10/22)	0.810 (94/116)	0.754 (104/138)	0.313 (10/32)	0.887 (94/106)
**External validation cohort** (*n* = 104)	DLS	0.852 (0.765–0.939)	0.667 (10/15)	0.798 (71/89)	0.779 (81/104)	0.357 (10/28)	0.934 (71/76)
	Non-transfer learning DLS	0.730 (0.584–0.877)	0.667 (10/15)	0.674 (60/89)	0.673 (70/104)	0.256 (10/39)	0.923 (60/65)

*Note* DLS, deep learning signature; AUC, area under curve; CI, confidence incidence; PPV, positive predictive value; NPV, negative predictive value

In addition, we employed 10-fold cross-validation, which redivided the training set into training and validation sets, to further evaluate the robustness of the proposed DLS method. The average results for the training set and validation set were 0.92 ± 0.01 and 0.89 ± 0.12, respectively. These results indicate that the model’s predictions did not exhibit significant fluctuations (Figure S2), demonstrating good robustness.

### MDLR model construction and verification

In constructing the MDLR model, this study used the size, NSE, pathological stage, CA199, and DLS as input variables for ELM classification. Therefore, we performed a collinearity analysis on these variables. The collinearity statistics showed that the size (Variance Inflation Factor (VIF) = 1.251), NSE (VIF = 1.079), pathological stage (VIF = 1.176), CA199 (VIF = 1.053), and DLS (VIF = 1.121) did not exhibit high correlations. All input variables passed the multicollinearity test (VIF < 10).

**Fig. 5:**
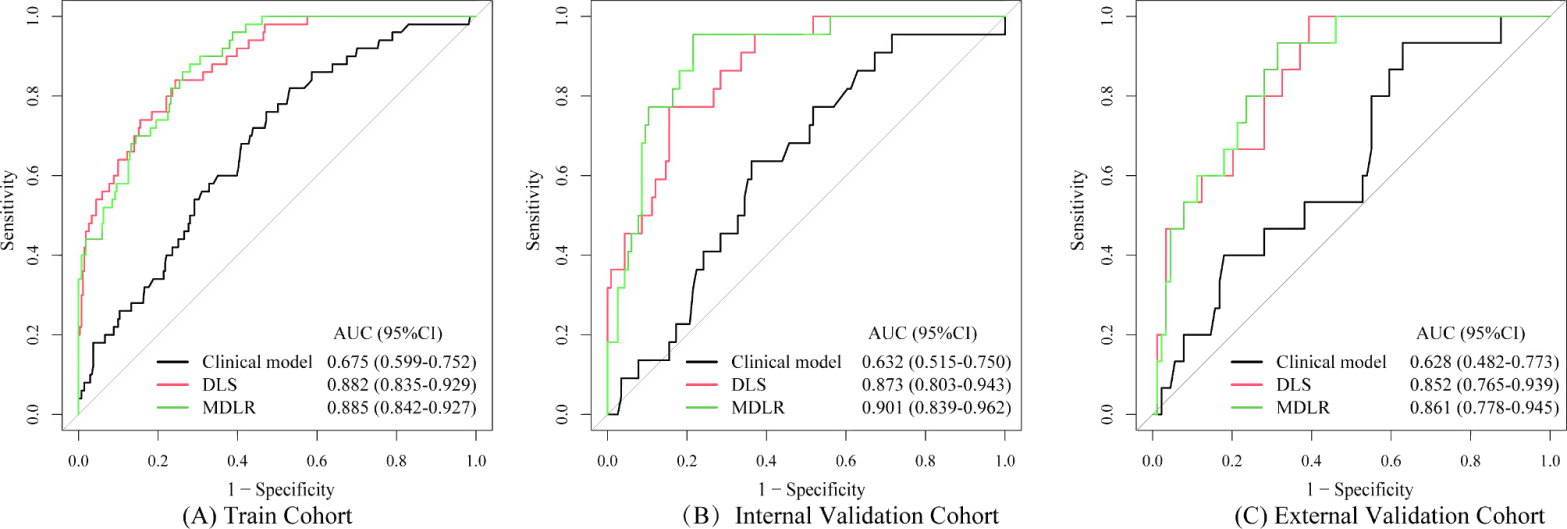
Receiver operating characteristic curves of the training cohort (**A**), internal validation cohort (**B**), and external validation cohort (**C**). MDLR, multimodal deep learning radiomics; DLS, deep learning signature; AUC, area under the curve; CI, confidence interval

In the training cohort, The AUC of the MDLR model in the training cohort was higher (AUC = 0.885 (95% CI: 0.842–0.927) than that of the clinical model and DLS model. In the internal validation cohort, the AUC of the MDLR model was 0.901 (95% CI: 0.839–0.962), higher than that of the clinical model (AUC = 0.632; 95% CI: 0.515–0.750) and DLS model (AUC = 0.873; 95% CI: 0.803–0.943). In the external validation cohort, the AUC of the MDLR model was 0.861 (95% CI: 0.778–0.945), higher than that of the clinical model (AUC = 0.628; 95% CI: 0.482–0.773) and DLS model (AUC = 0.852; 95% CI: 0.765–0.939) (Table 2; Fig. 5). DeLong tests were performed to compute the statistical differences between the MDLR, clinical model, and DLS. There was significant difference between MDLR and the clinical model (*p <* 0.001; *p <* 0.001; *p =* 0.003), but no significant difference between MDLR and DLS (all *p >* 0.05) in the training, internal and external validation cohorts, respectively (Table 4).

**Table 4:** Model evaluation improvement sheet

Datasets	Model 1 Model 2	DLRM
Training cohort	DLS	0.231 (*p* = 0.817)
	CM	4.849 (*p* < 0.001)
Internal validation cohort	DLS	0.800 (*p* = 0.424)
	CM	4.394 (*p* < 0.001)
External validation cohort	DLS	0.473 (*p* = 0.636)
	CM	2.946 (*p* = 0.003)

*Note* CM, clinical model; DLS, deep learning signature; MDLR, multimodal deep learning radiomics

Additionally, we used the threshold value corresponding to the maximum Youden’s index as the optimal diagnostic threshold. For each model, we continuously calculated the accuracy and other metrics for the training, internal validation, and external validation cohorts using the optimal diagnostic threshold determined from the training cohort. The optimal diagnostic thresholds for the clinical model, DLS, and MDLR in the training cohort were 0.144, 0.384 and 0.219, respectively. The diagnostic performance of each model at the unified threshold is presented in Table 2.

### Stratification analysis and decision curve analysis (DCA)

Stratified analysis was performed by gender, age, and scanning equipment. According to the above stratified analysis results, the *P* values of the Delong test were all greater than 0.05, indicating no statistical significance, indicating that the MDLR model established in this study had stable predictive performance for the postoperative progression of solid stage I NSCLC and was not affected by age, gender or equipment factors (Figure S1; Supplementary S7).

**Fig. 6:**
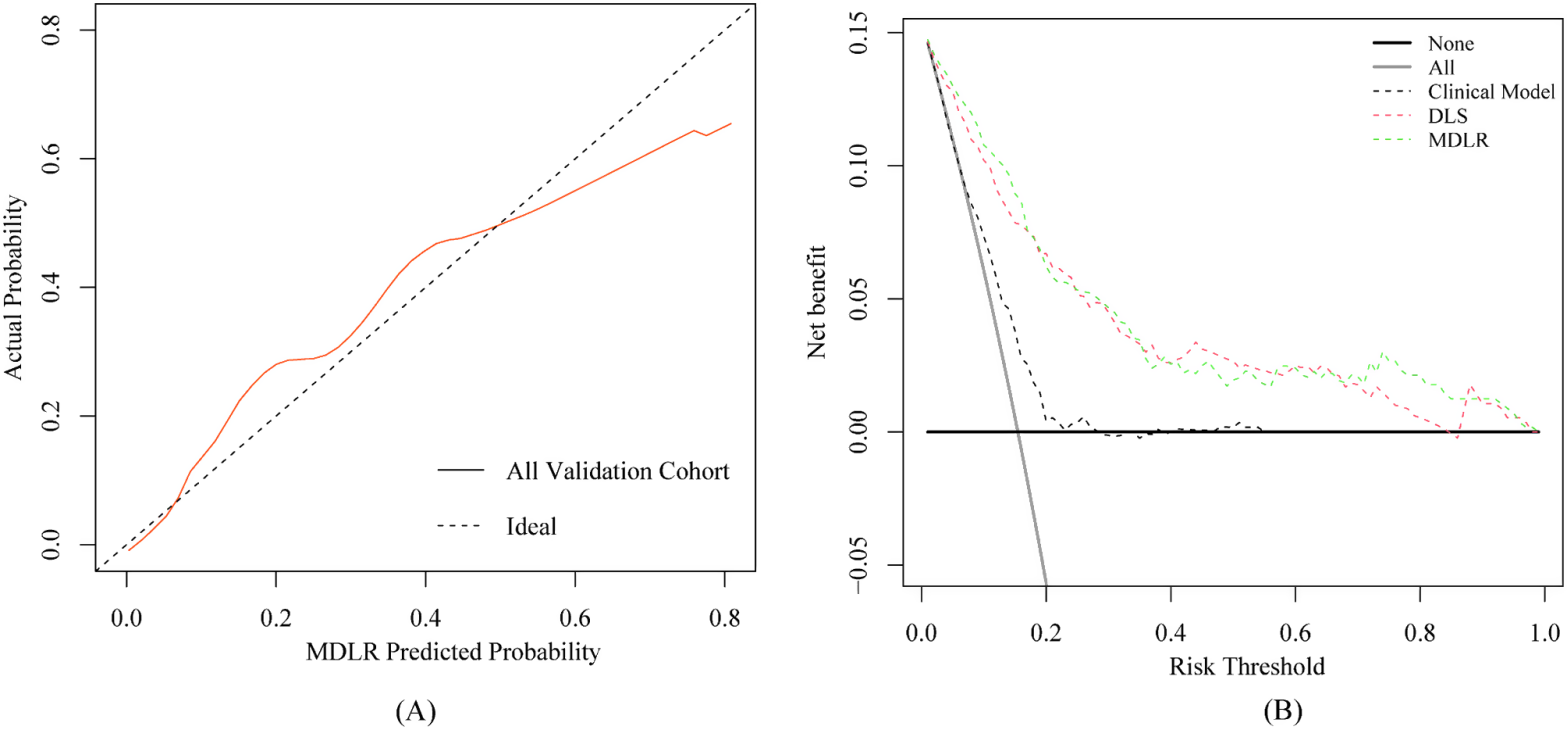
Correction curves and decision curve analysis. (**A**) the red curve is the correction curve for all the verification queues, and the dashed line is the correction curve under ideal conditions; (**B**) the solid black and grey lines indicate the assumption that all and none of the progression groups were involved, respectively; the threshold probability was defined as the point at which the expected benefit of the treatment was equal to the benefit of avoiding treatment; the results indicated that the MDLR model provided a greater net benefit than the clinical model and DLS (range 0.01–0.96). MDLR, multimodal deep learning radiomics; DLS, deep learning signature

DCA demonstrated that the MDLR model outperformed both the DLS model and the clinical model in terms of net benefits, with threshold probabilities ranging from 0.01 to 0.96 (Fig. 6**).**

## Discussion

In this study, clinical pathological factors and DL features were combined to establish a MDLR model for predicting the risk of postoperative progression of solid stage I NSCLC that could more comprehensively reflect tumour heterogeneity. The MDLR model had the best AUC of 0.885, superior to the clinical model (AUC = 0.675, delong test *p* < 0.001) and slightly better than the DLS model (AUC = 0.882, delong test *p* = 0.817). This MDLR model successfully divided the patients with solid stage I NSCLC into a high-risk group and a low-risk group, enabling better assessment of the risk of recurrence and metastasis after surgery to guide the postoperative follow-up and treatment of patients and develop personalized follow-up and treatment plans for patients.

Serum tumour markers are inefficient in the early diagnosis of NSCLC, but from a series of studies, scholars have reported the prognostic importance of several serum tumour markers [25–27]. Chen et al. evaluated the relationship between commonly used serum tumour markers and the recurrence of lung adenocarcinoma and squamous cell carcinoma. The results showed that preoperative elevation of serum CA199 predicted poorer relapse-free survival in patients with lung squamous cell carcinoma (*p* = 0.004) [28]. Our study investigated whether CA199 was an independent risk factor for postoperative progression of solid stage I NSCLC (*p* = 0.003), with results similar to those of the above study. Pathological stage was another independent risk factor for prognosis. TNM stage was the most common and important method to predict the overall survival rate. The median survival times of patients with pathologically staged stages IA, IIA and IIIA were 119 months, 49 months and 22 months, respectively, and the 5-year overall survival rates were 73%, 46% and 24%, respectively. The results demonstrated that there was a certain difference in prognosis between different pathological stages [5]. Most previous studies on the prognosis of lung cancer did not involve stratification by stage, and the results of the postoperative progression of lung cancer are quite different, ranging from 18-34.4% [4]. However, only patients with stage I lung cancer were included in our study, making it conducive to the realization of stage stratified management of NSCLC patients. In the current study, the postoperative progression rate was 15.7% (72/459) in center 1, which was similar to 14.4% (15/104). The recurrence rates of stage I NSCLC patients in the two centers were similar. Hung et al. [29] evaluated the prognostic predictors of postrecurrence survival in patients with resected stage I NSCLC, and the recurrence rate was 13.2% (123/933), which is very similar to our results.

Traditional machine learning has been used to predict the postoperative progression of NSCLC. Coroller et al. used traditional machine learning features to predict the distant metastasis of lung cancer [30]. Based on segmental CT images of 182 lung cancer patients, they utilized the minimum redundancy maximum relevance (mRMR) algorithm to screen 635 traditional machine learning features. Key features were selected by the clinical Cox regression model, and the results showed that 35 traditional machine learning features had high clinical diagnostic value for distant metastasis, 12 traditional machine learning features had considerable value for survival, and the accuracy of traditional machine learning features in predicting distant metastasis of lung cancer was 82%. However, traditional machine learning has great limitations, such as manual delineation of ROIs, specialized knowledge and complex task-specific optimization, and parameters have a great influence on feature extraction. In this study, we adopted the DL method to build the model. As an integrated classification model, DL has the following advantages: (1) image preprocessing does not need to accurately segment tumour lesions but only needs to frame and select the region of the lesions; (2) DL is based on data-driven learning, and the extracted DL features are more relevant to tasks; and (3) DL can save time and reduce errors caused by subjective factors associated with clinicians. In general, as a tool to assist clinicians, DL can reduce clinical pressure and exhibits higher diagnostic efficiency than traditional machine learning.

In this study, we developed a MDLR model aimed at identifying the progression status of solid stage I NSCLC and providing valuable insights for the treatment and follow-up of stage I NSCLC patients. Within the MDLR model, deep learning algorithms were employed to extract features from venous-phase images, and a sparse Bayesian ELM algorithm was utilized to classify these deep learning features. This approach served as the basis for the classification predictions of deep learning algorithms on venous-phase CT images of NSCLC patients. To comprehensively consider the disease status of NSCLC, we combined clinical and pathological factors with the DLS for analysis and modelling. Pathological stage, CA199, NSE, size, and DLS were identified as five factors with significant differences, and a sparse Bayesian ELM algorithm was utilized to construct the MDLR model. For reference and comparison, we evaluated the MDLR model against models that solely relied on DLS or clinical factors. The results indicated that the MDLR model algorithm outperformed the DLS and clinical models in all scenarios. Additionally, Delong’s test demonstrated statistically significant improvements in the MDLR model compared to the other two models. Therefore, the MDLR model approach can serve as a noninvasive preoperative predictive tool for assessing and forecasting the progression and prognosis of solid stage I NSCLC.

In clinical practice, for lung cancer nodules of the same size, the prognosis of solid nodules is considerably different from that of partially solid and nonsolid nodules. Hattori [31] evaluated the consolidation tumour ratio (CTR) in 1, 181 patients with surgically resectable NSCLC whose clinical stage was TxN0M0 and classified tumours into three groups, namely, the pure ground glass group (CTR = 0; *n* = 168), the partial solid group (0 < CTR < 1.0; *n* = 448) and the solid group (CTR = 1.0; *n* = 565). These results revealed that the 5-year overall survival times of nonsolid (pure ground glass density), partially solid and solid lung cancer patients were 100%, 94.6% and 75.4%, respectively. The recurrence risk of nonsolid lung cancer nodules and partial solid lung cancer nodules is low after surgery, and routine follow-up can be performed clinically. However, solid lung cancer nodules have a high risk of postoperative recurrence. Active intervention therapy, such as adjuvant chemoradiotherapy, is needed in clinical practice and needs to be studied further.

The limitations of this study are as follows: First, this study is retrospective and may have data selectivity bias. Prospective studies with larger samples are needed to confirm the stability and robustness of the diagnostic model in the future. Second, chest CT examination equipment models and scanning parameters were different, but we only selected patients with thin layer image thickness less than or equal to 1.5 mm. Third, during the process of deep learning feature extraction based on medical images, 3D images could provide deeper and more comprehensive features than 2D images. In the future, we will endeavor to incorporate 3D images to further refine our work and enhance the accuracy of our predictive model. Fourth, deep learning models are often regarded as “black-box” models, making it challenging to elucidate their decision-making processes. In the medical field, a comprehensive understanding of the model’s decision mechanism and reliability is crucial. Therefore, enhancing the interpretability of deep learning models is an issue that needs to be addressed.

## Conclusion

In conclusion, the MDLR model that incorporated clinicopathological characteristics, subjective CT findings and DLS showed a high performance in predicting postoperative progression of solid stage I NSCLC, which will facilitate the suitable treatment method selection and follow-up in patients with solid stage I NSCLC.

## Electronic supplementary material

Below is the link to the electronic supplementary material.

Supplementary Material 1

Supplementary Material 2

## Data Availability

The datasets used and/or analyzed during the current study are available from the corresponding author on reasonable request.

## Abbreviations

AUC: Area under curve
CA199: Carbohydrate antigen 19 − 9
CEA: Carcinoma embryonic antigen
CI: Confidence interval
CTR: Consolidation tumour ratio
CSP: Circumsporozoite protein
Cyfra21-1: Cytokeratin 19 fragment
DCA: Decision curve analysis
DFS: Disease free survival
DICOM: Digital imaging and communications in medicine
MDLR: Multimodal deep learning radiomics
DLS: Deep learning signature
ELM: Extreme learning machine
GGO: Ground glass opacity
LLL: Left lower lobe
LUL: Left upper lobe
LVI: Lymphovascular invasion
mRMR: Minimum redundancy maximum relevance
NCCN: National comprehensive cancer network
NPV: Negative predictive value
NSCLC: Non-small cell lung cancer
NSE: Neuron specific enolase
OR: Odds ratio
OS: Overall survival
PACS: Picture archiving and communication system
PPV: Positive predictive value
PRS: Post-relapse survival
RLL: Right lower lobe
RML: Right middle lobe
ROC: Receive operating curve
RUL: Right upper lobe
SD: Standard deviation
