# Supplementary material for: Multimodal deep learning radiomics model for predicting postoperative progression in solid stage I non-small cell lung cancer
Source: Cancer Imaging. 2024 Oct 17;24:140. doi: 10.1186/s40644-024-00783-8 (PMC11487701; doi:10.1186/s40644-024-00783-8)
Supplement: Supplementary file 1 — Supplementary Material 1 [file 40644_2024_783_MOESM1_ESM.docx]

**Supplementary Materials**

***S1. Chest CT scan techniques***

Chest CT was conducted by one of the following scanners: Definition Force (Siemens, German), Siemens 16 (Siemens, German), Toshiba Aqilion (Toshiba, Japan), and GE Discovery 64 (GE, USA). Spiral CT volume scanning technology was adopted, and the scanning parameters were as follows: tube voltage, 120 KVp; automatic tube current technology, 40-150 mAs; pitch, 0.75-1.0; matrix, 512×512; and FOV, 200 mm×200 mm; the scanning radiation dose was approximately 50-150 mGy; scans ranged from bilateral lung tips to the adrenal level; the patient was placed in the supine position, hands raised, and the scan was started with deep inspiration and breath holding; to obtain contrast-enhanced images, ioprotamine (350-370 mgI/mL) was injected intravenously with a power syringe at a rate of 2.5-3.5 mL/s, CT scans were performed 25 seconds (arterial phase) and 50 seconds (venous phase) after injection, and cross-sectional enhanced CT images of the venous phase were selected; the transverse position of the reconstructed image was 0.625-1.50 mm, with 20-30% overlap; coronal and sagittal images were 3.0 mm thickness.

***S2. Subjective CT finding evaluation***

CT findings evaluation was as follows: two radiologists (with 10 and 15 years of experience in chest imaging diagnosis) independently evaluated the CT findings of lung cancer nodules; subjective findings included emphysema, margin, lobulated sign, speculated sign, vacuole sign and air bronchogram sign; if there was disagreement, the CT findings were discussed together; transverse images of the largest layer of lung cancer nodules were selected for longest diameter measurement, and the average value measured by two doctors was finally taken as the longest diameter; other CT findings included location (left upper lobe (LUL), left lower lobe (LLL), right upper lobe (RUL), right middle lobe (RML), and right lower lobe (RLL)); the definition of subjective findings was as follows: lobulated sign—nodules or masses grow at different rates in all directions or are blocked by surrounding structures; the contour can be a plurality of arc-shaped protrusions, and the arc-shaped interphase is a concave notch, forming a lobed shape; speculated sign—several linear shadows of varying lengths with little difference in the thickness of the far and near ends extending from the edge of a nodule or mass to the surrounding lung parenchyma, not connected to the pleura; vacuole sign—gas density shadows or low-density shadows with a diameter of less than 5 mm can be single or multiple, and no low-density dot shadows can be seen on more than two adjacent layer; air bronchogram sign—large air bronchi are contrasted with solid lung tissue, with air bronchi branching shadows seen in the area of consolidation^[1]^.

***S3. Pathological stage and postoperative progression definition***

Stage I includes (T1-T2a) N0; in the T1 stage, the maximum tumour diameter was less than 3.0 cm. in stage T2a, the maximum diameter of the tumour was greater than 3.0 cm and less than 4.0 cm or had any of the following characteristics: (1) invasion of the main bronchus, but not into the carina; (2) invasion of visceral pleura; and (3) obstructive pneumonia involving hilum or partial or total atelectasis.

Definition of postoperative progress was as follows: (1) during postoperative follow-up, the presence or absence of recurrence or metastasis was comprehensively determined by physical examination, imaging methods and tumour marker detection; (2) local recurrence refers to lesions present at the operative margin, ipsilateral thorax or mediastinum; (3) distant metastasis refers to lesions in the contralateral lung or chest and organs other than the mediastinum; (4) if clinical conditions permit, the diagnosis should be confirmed by histopathological or cytological examination as far as possible; and (5) suspected relapsed or metastatic lesions not confirmed by histopathology, with a larger diameter during follow-up or smaller diameter after antitumour therapy, were clinically diagnosed as relapsed or metastatic; grouping—the positive group developed within 3 years; the negative group had no progression in 3 years^[2, 3]^.

***S4.*** ***Deep learning experimental equipment***

The segmentation of the tumor ROI region, we used custom programs written in MATLAB 2020b software, employing a 2D bounding box segmentation method.

The deep learning training environment was as follows: PyCharm version 2021.3 was used as the software development environment. An NVIDIA RTX A6000 graphics card with CUDA version 10.2 and 48 GB of GPU memory was utilized for accelerated computation. The deep learning framework PyTorch 1.7.1 with GPU support was employed, implemented in Python 3.6. Additionally, MATLAB version 2020b was used for feature selection tasks. Detailed parameters of deep learning training are shown in Supplementary S5.

***S5. Detailed parameters of deep learning training***

The parameter settings for the deep learning model are as follows: initial learning rate of 0.1, input sample batch size of 128, optimization algorithm using stochastic gradient descent with momentum, where the momentum term of the optimizer is set to 0.9, decay factor is 0.0001, and cross-entropy is used as the loss function to reflect the loss between the target and the expected values.

***S6. Deep learning model construction***

In this study, an ELM is used to construct a classification model. As a feed forward neural network with a single hidden layer, the network not only has a simple structure but also has excellent performance in learning speed and prediction accuracy. In the network, the connection weight and the offset of the activation function between the input layer and the hidden layer are randomly given, and the solution process is as follows:

If there are N arbitrary samples, among ,. A single hidden layer neural network with hidden
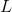
 layer nodes can be expressed as

. (1)

where is the activation function. is the input weight. is the output weight. is the bias of the hidden layer unit. represents the inner product of and . The goal of single hidden layer neural network learning is to minimize the output error, which can be expressed as

(2)

That is, there are two types of , and , which make

(3)

The matrix can be expressed as

(4)

where is the output matrix of the implicit layer, is the desired output, and is the output weight.

(5)

Among them, ,. To train the single hidden layer neural network, we hope to obtain , and to make

(6)

Among them, , which is equivalent to minimizing the loss function as follows:

(7)

***S7. Stratification analysis of MDLR***

A total of 563 patients with solid stage I NSCLC had an AUC of 0.870 (95% CI: 0.837-0.903) based on the training cohort (321 cases), internal validation cohort (138 cases) and external validation cohort (104 cases). Stratified analysis was performed by gender, age, and equipment.

Among all the data, the AUC for male patients (n = 293) was 0.875 (95% CI: 0.831-0.919), p = 0.870 for the Delong test; the AUC for female patients (n = 270) was 0.864 (95% CI: 0.813-0.915), p =0.841 for the Delong test.

In the age stratification analysis, 60 years old was used as the dividing line. The diagnostic efficiency of age ≤ 60 years (n = 280) was 0.884 (95% CI: 0.841-0.928), and the Delong test p = 0.621. The diagnostic efficiency in patients older than 60 years (n = 283) was 0.859 (95% CI: 0.809-0.910), and the Delong test p = 0.721.

Stratified analysis of the four devices showed that the diagnostic efficiency of the Siemens 16-slice CT (n = 81) was 0.851 (95% CI: 0.750-0.952), and that of the Delong test was p = 0.718. The Toshiba Aqilion 64-row CT (n = 192) diagnostic efficiency was 0.903 (95% CI: 0.857-0.950), Delong test p = 0.254. The definition force (n = 240) diagnostic efficiency was 0.852 (95% CI: 0.792-0.911), Delong test p = 0.594. The GE Discovery 64-slice CT (n = 50) diagnostic efficiency was 0.856 (95% CI: 0.744-0.969), Delong test p = 0.816.

According to the above stratified analysis results, the *P* values of the Delong test were all greater than 0.05, indicating no statistical significance, indicating that the MDLR established in this study had stable predictive performance for the postoperative progression of solid stage I NSCLC and was not affected by age, gender or equipment factors. Figure S1 shows the ROC curve for hierarchical analysis of gender, age, and equipment.

**References:**

1. Koo HJ, Xu H, Choi CM, et al. Preoperative CT Predicting Recurrence of Surgically Resected Adenocarcinoma of the Lung. Medicine (Baltimore). 2016; 95(2):e2513.

2. Toba H, Kawakita N, Takashima M, et al. Diagnosis of recurrence and follow-up using FDG-PET/CT for postoperative non-small-cell lung cancer patients. Gen Thorac Cardiovasc Surg. 2021; 69(2):311-17.

3. Shimada Y, Saji H, Yoshida K, et al. Prognostic factors and the significance of treatment after recurrence in completely resected stage I non-small cell lung cancer. Chest. 2013; 143(6):1626-34.
