# Supplementary material for: Multimodal deep learning radiomics model for predicting postoperative progression in solid stage I non-small cell lung cancer
Source: Cancer Imaging. 2024 Oct 17;24:140. doi: 10.1186/s40644-024-00783-8 (PMC11487701; doi:10.1186/s40644-024-00783-8)
Supplement: Supplementary file 2 — Supplementary Material 2 [file 40644_2024_783_MOESM2_ESM.docx]

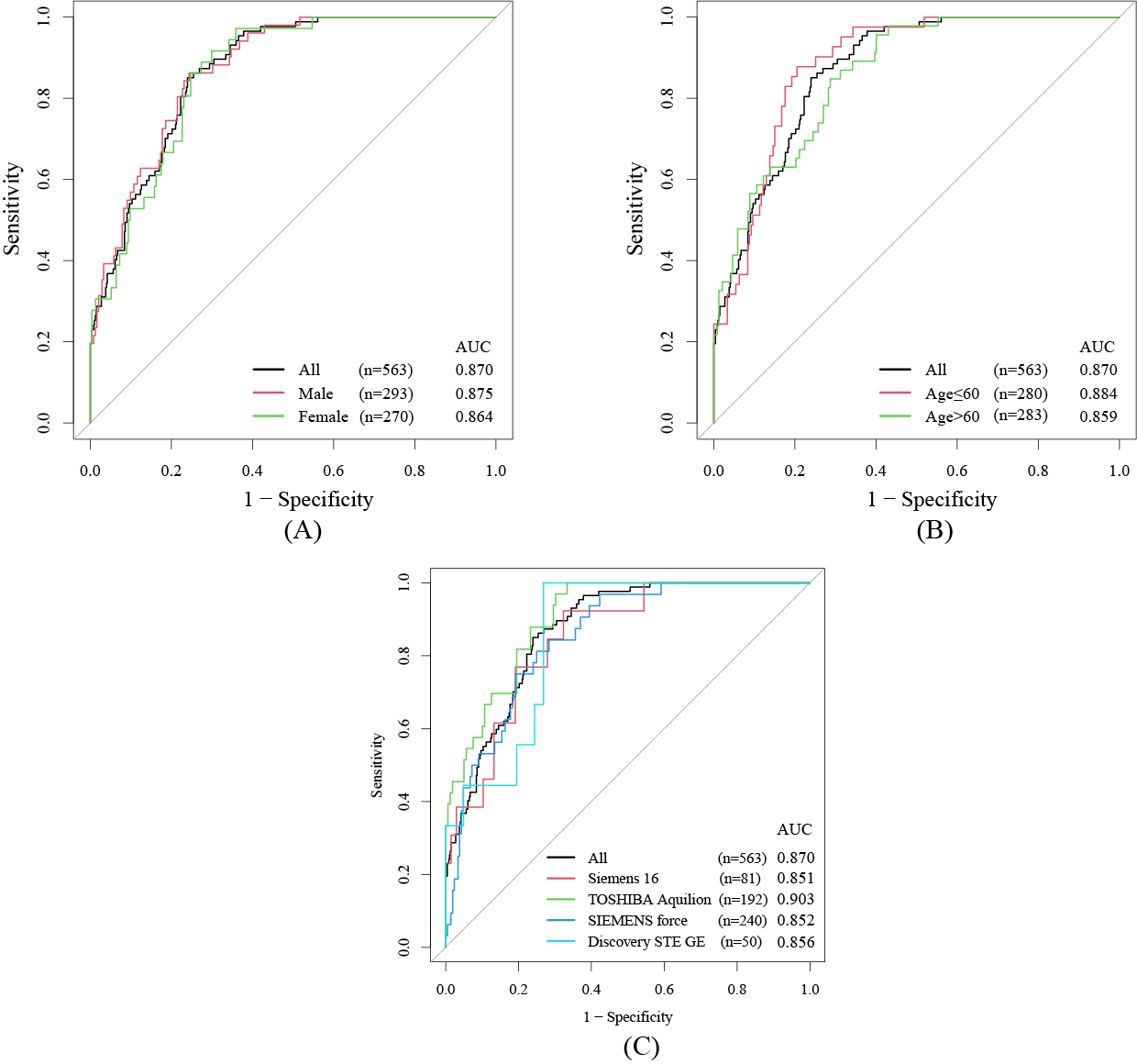


**Figure S1.** Stratified analysis of the diagnostic effectiveness of MDLR model.

Gender (A), age (B) and CT scanning equipment (C) were verified respectively. MDLR, multimodal deep learning radiomics; AUC, area under the curve.


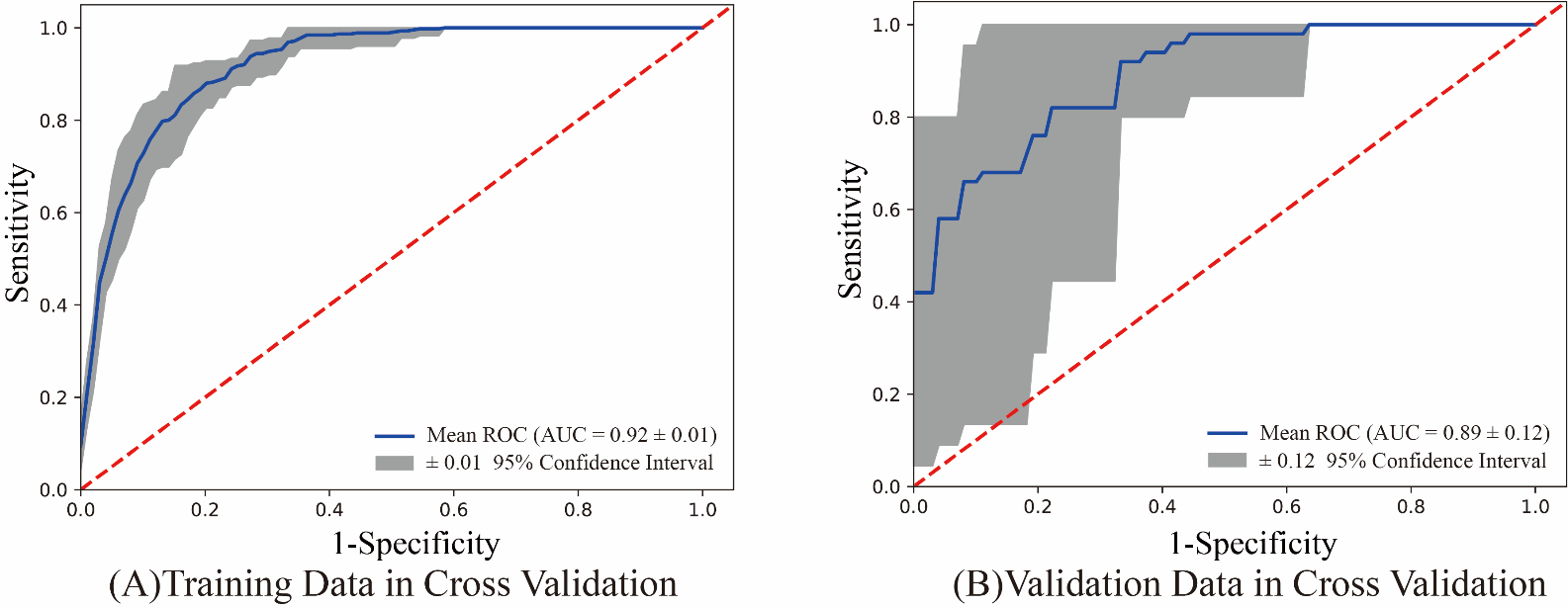


**Figure S2.** ROC curves for the DLS model using 10-fold cross-validation.
